# Microbial denitrification characteristics of typical decentralized wastewater treatment processes based on 16S rRNA sequencing

**DOI:** 10.3389/fmicb.2023.1242506

**Published:** 2023-09-14

**Authors:** Shanqian Huang, Yaping Kong, Yao Chen, Xuewen Huang, Pengfei Ma, Xuexin Liu

**Affiliations:** ^1^Center of Environment Protection, China Academy of Transportation Sciences, Beijing, China; ^2^Anhui Transportation Holding Group CO., LTD., Hefei, China; ^3^Qinghai Expressway Maintenance Service CO., LTD., Xining, China

**Keywords:** 16S rRNA, multi-media biofilter, decentralized wastewater treatment, denitrification flora, LEfSe, FAPROTAX

## Abstract

Despite the widespread application of decentralized wastewater treatment (WWT) facilities in China, relatively few research has used the multi-media biological filter (MMBF) facilities to investigate the microorganism characteristics. This study utilizes 16S rRNA high-throughput sequencing (HTS) technology to examine the microbial biodiversity of a representative wastewater treatment (WWT) system in an expressway service area. The pathways of nitrogen removal along the treatment route were analyzed in conjunction with water quality monitoring. The distribution and composition of microbial flora in the samples were examined, and the dominant flora were identified using LEfSe analysis. The FAPROTAX methodology was employed to investigate the relative abundance of genes associated with the nitrogen cycle and to discern the presence of functional genes involved in nitrogen metabolism. On average, the method has a high level of efficiency in removing COD, TN, NH3-N, and TP from the effluent. The analysis of the microbial community identified a total of 40 phyla, 111 classes, 143 orders, 263 families, and 419 genera. The phyla that were predominantly observed include *Proteobacteria, Acidobacteria*, *Chloroflexi*, *Actinobacteria*, *Nitrospirae*, *Bacteroidetes*. The results show that the system has achieved high performance in nitrogen removal, the abundance of nitrification genes is significantly higher than that of other nitrogen cycle genes such as denitrification, and there are six nitrogen metabolism pathways, primarily nitrification, among which Nitrospirae and Nitrospira are the core differentiated flora that can adapt to low temperature conditions and participate in nitrification, and are the dominant nitrogen removal flora in cold regions. This work aims to comprehensively investigate the diversity and functional properties of the bacterial community in decentralized WWT processes.

## 1. Introduction

Decentralized wastewater treatment (WWT) facilities are prevalent in Japan, North America, and Europe (Dueholm et al., 2022), and are regularly utilized in China for transportation facilities such as expressway service areas, water service areas, and marine vessels (Starkl et al., 2015). While onshore water treatment technologies are commonly used for the domestic WWT process in vessels, recent research frequently utilizes municipal domestic sewage, primary sedimentation pool effluent, and similar sources instead of vessels’ domestic sewage for experimental investigations (Yin et al., 2019; Yasir, 2020; Zhang et al., 2020; Rodriguez et al., 2021). In contrast to municipal-scale WWT facilities, WWT facilities designed for vessels’ domestic wastewater and expressway service areas exhibit distinct characteristics. These include significant fluctuations in water volume, a substantial pollution burden, elevated levels of nitrogen and phosphorus, and a low carbon-to-nitrogen ratio. Moreover, the selection of treatment processes for these facilities should consider the impact on environmental conditions. Additionally, it is essential to consider the benefits of cost reductions, energy conservation, and manageable maintenance when designing such facilities.

Biological nitrogen removal, which is a WWT process driven by microorganisms, has emerged as the predominant technology for nitrogen removal in transportation services. This is mostly attributed to its notable efficiency, cost-effectiveness, and absence of secondary pollutants. Nevertheless, it is crucial to note that microorganisms exhibit optimal performance within a temperature range of 20–35°C (Jankowski et al., 2022). In circumstances of temperatures falling below this range, microbial activity is restricted which consequently affects the performance of biochemical treatment. Therefore, the majority of decentralized WWT processes implemented in transport service facilities interact with challenges in meeting standard emission requirements during the winter season. Recently, there have been extensive studies conducted to enhance the aeration biofilter WWT process. These studies concentrated on various aspects including the development of novel biological carriers, the implementation of exclusive microbial reinforcement, and the incorporation of thermal insulation. Consequently, a novel technology known as the multi-media biological filter (MMBF) WWT was developed, which has been successfully applied in several practical scenarios. The utilization of high-capacity burden, space optimization, reliable operation, improved effluent quality, efficient management, and cost-effectiveness are among the notable advantages (Yang et al., 2021).

The removal rates of pollutants in biochemical treatment systems are typically influenced by the composition of microbial flora (Kristensen et al., 2020). As a consequence, the structure of microbial flora plays an essential part in maintaining the stability of WWT systems. Therefore, investigating the characteristics of microbial flora is crucial for comprehending and forecasting the performance of pollutant removal and degradation metabolism functions. At present, there is a limited number of studies that have employed the MMBF in WWT systems. However, it is significant to consider that the microbial community structure plays a vital role in ensuring the stability of these systems. In addition, it is noteworthy that the influence of various treatment methods on the microbial community structure demonstrates an extent of variability. Therefore, it’s beneficial to explore the attributes of the microbial and microorganism community structure in conventional decentralized WWT systems. This endeavor aims to enhance the treatment process, facilitate the dynamic adjustment of maintenance and management approaches, and confirm the efficiency of treatment.

Technologies such as Illumina Miseq and other high-throughput sequencing (HTS) have undergone rapid development and have been extensively utilized in the analysis of microbial communities in large-scale ecosystems and global WWT plants (Fernandez-Cassi et al., 2018; Rodriguez et al., 2021). These technologies have been employed to investigate microbial functional genes that play a crucial role in material cycling. Consequently, they serve as valuable instruments for exploring the composition and dynamics of microbial community structures in WWT processes. Additionally, they facilitate the identification of functional microflora and microbial genes, as well as the exploration of interactions among microorganisms. Microbial investigations conducted on WWT systems have revealed that the microbial communities present in domestic wastewater exhibit notable distinctions from those found in industrial wastewater, as well as from microbial communities inhabiting air, freshwater, marine, and soil habitats (Zhang et al., 2018). Wastewater features display great diversity, typically manifesting various bacterial community compositions that are representative of various functions (Yang et al., 2020b). The 16S rRNA HTS technology (Dueholm et al., 2022) is commonly used to analyze the composition and diversity of microorganisms in different environments (Jing et al., 2021). By utilizing specific nucleic acid fragments as biomarkers, this sequencing technology has become widely adopted for quantifying functional microorganisms, including nitrifying bacteria, denitrifying bacteria, and Anammox bacteria in activated sludge systems (Lukumbuzya et al., 2020). Additionally, this technology enables the investigation of the microflora structure at various taxonomic levels, such as kingdom, phylum, order, family, genus, and species, in samples from different sources. The characterization of microbiological samples can be achieved by the utilization of various indices such as Shannon, Simpson, Chao, and Ace. These indices offer insights into the structural properties of the microbial community, the relative abundance of functional genes, and the taxonomic affiliation of the species within the microbial samples (Cinà et al., 2019).

The primary objectives of this research are to investigate the variability in microbial flora structure and identify the key organisms involved in denitrification processes within the system. This study involves the collection of microbial samples from MMBFs located in expressway service areas. The samples are then subjected to 16S rRNA HTS to analyze the composition of the microbial community. Additionally, the study investigates the presence of functional genes associated with nitrogen metabolism. The main objective is to comprehensively investigate the diversity and functional traits of microbial flora in decentralized WWT processes. The preliminary results of this research attempt to provide theoretical and empirical evidence to promote the development of efficient decentralized biological treatment systems for wastewater.

## 2. Materials and methods

### 2.1. Sampling of water quality

#### 2.1.1. Selection of typical processes

**Figure 1:**
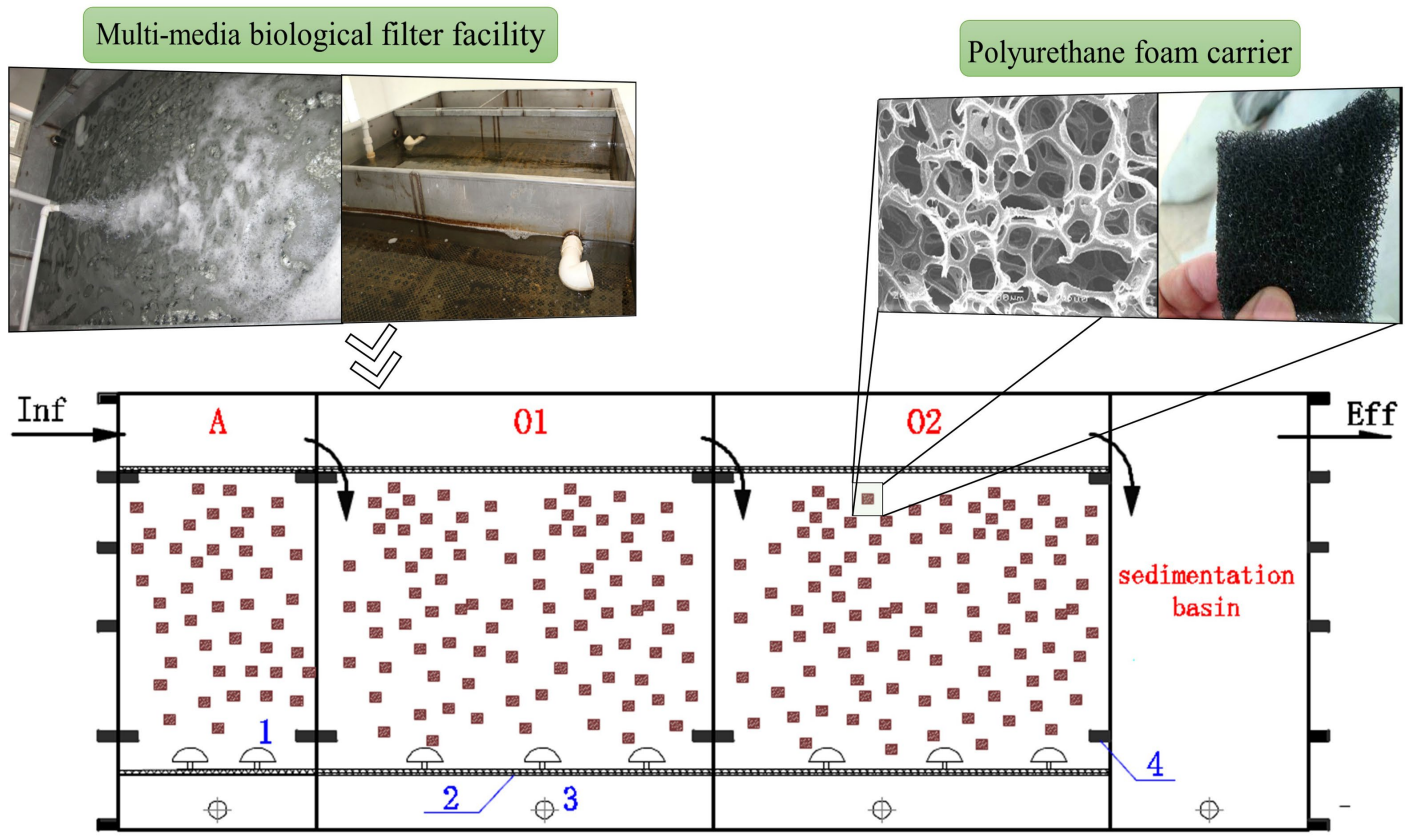
Application of MMBF process (numbers in blue: 1-air pipe, 2-outlet screen, 3-sludge pipe, 4-reinforced square pipe). The facility is consist of anoxic cell (A) using micro-aeration, aerobic cell 1 (O1) and aerobic cell 2 (O2) using regular aeration.

The MMBF process was selected for this study from an expressway service area in Jilin Province, China. The facility was initiated in 2014 with a designed treatment capacity of 60 m^3^/d which had been operating stably since then, with the average water temperature maintained at 15–23°C. As shown in Figure 1, the process utilized a sequential inlet and aeration intermittently, consisting of anoxic cell (A), aerobic cell 1 (O1) and aerobic cell 2 (O2). The anoxic cell of MMBF utilized micro-aeration, while the aerobic cells utilized regular aeration, with the aeration time of 10-15 min/h under stable operation. In addition, the cells filled with functionalized polyurethane foam carriers, with inoculation of high efficiency microbial organisms initially. During operation, sludge was removed every 2 to 3 years to maintain stable sludge concentration in the system.

#### 2.1.2. Sample collection and measurement methods

To investigate the pollutant removal effect of the MMBF, water samples were collected quarterly from the A, O1 and O2 cells over the stable operation phase between 2014 and 2019. The sampling sites were located at the terminal, inlet and outlet of each cell in the MMBF. Following the filtration, the concentrations of pH, chemical oxygen demand (COD), total nitrogen (TN), NH_3_-N, total phosphorus (TP), suspended solids (SS) and other indicators were measured *in situ*. In addition, to further analyze the effect of nitrogen removal along the process, samples from 2020 were collected to measure NO_3_^−^-N, NO_2_^−^-N and other indicators concentrations. The pH was measured by glass electrode method, COD by dichromate method, TN by ultraviolet spectrophotometer with alkaline potassium persulphate, NH_3_-N by salicylic acid spectrophotometer, TP by ammonium molybdate spectrophotometer, NO_2_^−^-N by N-(1-Naphthyl)-Ethylenediamine spectrophotometry and NO_3_^−^-N by thymol spectrophotometry (Koistinen et al., 2019).

### 2.2. Sequencing of microbial samples

#### 2.2.1. Microbial sample collection

Considering the progressive character of the WWT process, it is recommended to establish an appropriate sampling interval in order to ascertain the precision of the collected data. In this study, a total of nine sampling sites were randomly selected from the upper (ID:X1), middle (ID:X2), and lower (ID:X3) layers within each cell. These specimens are identified as cell A (sample ID: A1, A2, A3), cell O1 (sample ID: B1, B2, B3), and cell O2 (sample ID: C1, C2, C3). The specimens were obtained and delivered to the laboratory using cryogenic storage techniques (below 0°C). Subsequently, the genomic DNA was extracted and preserved at the temperature of −20°C.

#### 2.2.2. Gene extraction and HTS

The specimens were centrifuged and well mixed subsequent to their melting on ice. Afterward, the quality and concentration of the extracted DNA were assessed by Nano Drop 2000 spectrophotometer (Thermo Scientific). All specimens were subjected to this procedure under standardized experimental conditions in Allwegene lab, with three replicates performed for each specimen. The extracted DNA, amounting to 30 ng, was then utilized for polymerase chain reaction (PCR) using TransGen AP221-02. The amplification of the V3-V4 region of the bacterial 16S rRNA gene was carried out using the TransStart Fastpfu DNA Polymerase, with the application of universal primers sequence 5′-3′: ACTCCTACGGGAGGCAGCAG (Cannon et al., 2019). The PCR outputs derived from the identical specimen were combined and subjected to analysis in order to assess the purity and integrity of the DNA (Yang et al., 2020a). This analysis was carried out using 1% agarose gel electrophoresis technique. Then, the 16S rRNA V3-V4 region was sequenced on the Illumina Miseq PE300 sequencing platform to obtain high-throughput gene data (Liu Y. et al., 2020).

#### 2.2.3. Annotation of gene function

After the data obtained from high throughput sequencing completed assembly and quality filtering, the filtered 16SrRNA gene sequences were submitted to further analysis using the QIIME platform (Bolyen et al., 2019). To ensure data quality, TRIMMOMATIC (Bolger et al., 2014) and PEAR were utilized for data quality control, specifically for the removal of low-quality sequences. Subsequently, optimized sequences were obtained through sequence assembly, filtration, and chimerization processes utilizing FLASH, PEAR, and UCHIME. According to the UPARSE method (Edgar, 2013) and UNOISE3 method (Rognes et al., 2016), the sequences performed clustering and noise reduction processes to generate operational taxonomic units (OTUs), using a similarity criterion of 97%. In the comparative analysis of OTU representative sequences, the taxonomic annotation of OTUs at all levels (kingdom, phylum, class, order, family, genus, species) was performed using the RDP Classifier, UCLUST, and the SILVA database (Quast et al., 2012; Zhang et al., 2019a).

### 2.3. Methods of data analysis

#### 2.3.1. Measurement on relative abundance of species

In order to identify the microbial species and relative abundance in the specimens, as well as to investigate variations in community structure such as species composition and bacterial concentration at different taxonomic levels (Zhang et al., 2019b), clusters were generated using the R language. These clusters were based on the Unweighted Unifrac distance matrix, and utilized the UPGMA method to analyze the specimens and the genera present at the phylum level. The clustering outcomes were visually represented by displaying the relative abundance of each specimen. Heatmaps were generated at the phylum and genus levels to illustrate the variations in community structure across different levels of the genus, based on the abundance of OUT in each specimen.

#### 2.3.2. Analyses on community diversity

To evaluate the diversity of microorganisms within the context of community ecology, the analysis of alpha diversity was performed. This analysis utilized the QIIME platform (Bolyen et al., 2019) to evaluate the abundance and diversity of microbial communities at the operational taxonomic unit (OTU) level. Various statistical indicators, such as observed species, goods coverage, Shannon index, and Chao1 index, were calculated to examine community abundance, diversity, and coverage, in order to identify species that exhibited variability (Liu Y. et al., 2020). Within this set of indicators, Chao1 is defined as the species abundance indicator, estimating the number of OTUs in the community. Observed species, on the other hand, represent the count of OTUs observed as sequencing depth increases. The goods coverage metric describes the extent to which each specimen database is covered, thereby reflecting the likelihood of detecting sequences within the specimen. Additionally, the Shannon index is applied to assess the diversity of microorganisms within the specimen.

#### 2.3.3. Analyses on core divergent flora

To investigate significantly distinct species serve as biomarkers among different groups based on their abundance, LDA Effect Size (LEfSe) (Segata et al., 2011) was implemented using the R language MICROECO package (Liu C. et al., 2020). The analysis were performed on nine specimens from three cells, utilizing a non-parametric factorial Kruskal-Wallis hierarchical test to identify species with significant differences in abundance across different subgroups. Subsequently, a Wilcoxon hierarchical test was applied to analyze differences within groups. Ultimately, a linear discriminant analysis (LDA) was conducted to reduce the dimension of the data and evaluate the impact of relevant species, as indicated by the LDA score. In this study, a threshold value of 4 was used to determine the significance of the LDA score.

#### 2.3.4. Prediction on the function of the bacterium community

To explore the functional diversity of the bacterial microbial community in the water following WWT, a functional prediction analysis was conducted using the FAPROTAX method (Louca et al., 2016). This method was recognized as high prediction accuracy based on validated literature on culturable bacteria, available for functional prediction of the nitrogen cycle in environmental specimens (Chen et al., 2018). The analysis utilized a classification table of operational taxonomic units (OTUs) based on 16S sequencing, which allowed for the identification of microbial genera and species names. Consequently, the analysis provided annotated predictions of microflora function, which was performed by Tutools platform.^1^

## 3. Results

### 3.1. Characteristics of water quality

#### 3.1.1. Pollutant removal results

Due to significant variations in pedestrian flow at the service area and seasonal differences in water consumption, the influent water quality fluctuates considerably. As a result, the effluent concentrations of COD range from 298.2 to 720.0 mg/L, TN ranges from 48.65 to 108.71 mg/L, and NH_3_-N ranges from 15.4 to 152.00 mg/L. As shown in Table 1, the COD removal ratio ranges from 84.98 to 97.83%, the NH_3_-N removal ratio ranges from 92.23 to 97.78%, and the TP removal ratio ranges from 88.86 to 96.50%. The effluent’s average COD, TN, NH_3_-N and TP concentrations were measured at 28.62, 13.8, 3.17, and 0.66 mg/L, respectively. These values were confirmed in compliance with the applicable standards of regulations. In summary, this process high efficiency in mitigating nitrogen, COD, and TP levels in the wastewater originating from the service area. Moreover, it exhibits remarkable resilience towards fluctuations in load, contributing to stable effluent quality.

**Table 1:** The average results of water quality indicators (unit: mg/L) and contaminants removal (%) quarterly detected in the inlet and outlet of MMBF.

Index	pH	COD	TN	NH_3_-N	TP	SS
Inf	6.87 ± 0.77	491.34 ± 211.11	76.55 ± 30.26	58.36 ± 48.8	6.8 ± 2.81	58.73 ± 44.87
Eff	7.38 ± 0.46	28.62 ± 14.92	13.8 ± 4.59	3.17 ± 2.51	0.66 ± 0.3	2.57 ± 0.81
Removal (%)		0.93 ± 0.06	0.78 ± 0.15	0.92 ± 0.1	0.89 ± 0.04	0.95 ± 0.03

#### 3.1.2. Nitrogen removal pathway

**Figure 2:**
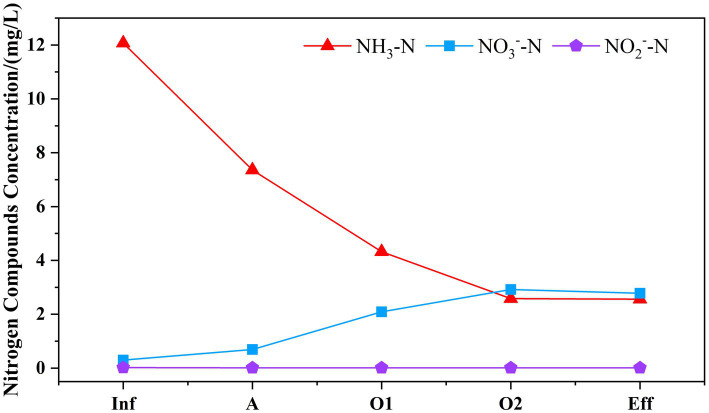
Variation of nitrogen removal pathways along the MMBF system. The variation of NH_3_-N, NO_3_^−^-N, NO_2_^−^-N was displayed in different color and bars to reflect the variation of nitrogen concentration along the system.

As presented in Figure 2, the concentrations of NH_3_-N, NO_3_^−^-N, NO_2_^−^-N in the effluent were measured as 2.56, 0.01, 2.78 mg/L, respectively. Based on the assessment of nitrogen conversion, the system eliminated 14.41 mg/L of TN and 9.51 mg/L of NH_3_-N, with no traces of nitrite detected throughout the process. The percentage contributions to TN removal in cell A, O1, and O2 were 68.36, 18.67, and 13.95% respectively, suggesting that the primary removal of TN occurred in cell A. The existence of anaerobic ammonia oxidation (anammox) is postulated. The primary pollutant NH_3_-N present in the wastewater was initially imported into the anoxic cell of the system, resulting in the removal of 4.71 mg/L. This removal might be attributed to the partial conversion of NH_3_-N to NO_3_^−^-N through nitrification in the micro-aerobic environment, followed by further conversion to N_2_ through denitrification, resulting in the removal NO_3_^−^-N of 4.32 mg/L. Additionally, it facilitates the accumulation of anaerobic ammonia bacteria. When wastewater flows into the aerobic cell, the NH_3_-N is oxidized further through regular aeration resulting in a significant reduction in the TN concentration, which facilitates the effluent water quality to meet the standard.

### 3.2. Characteristics of the predominant flora

#### 3.2.1. Characterisation on phylum level

**Figure 3:**
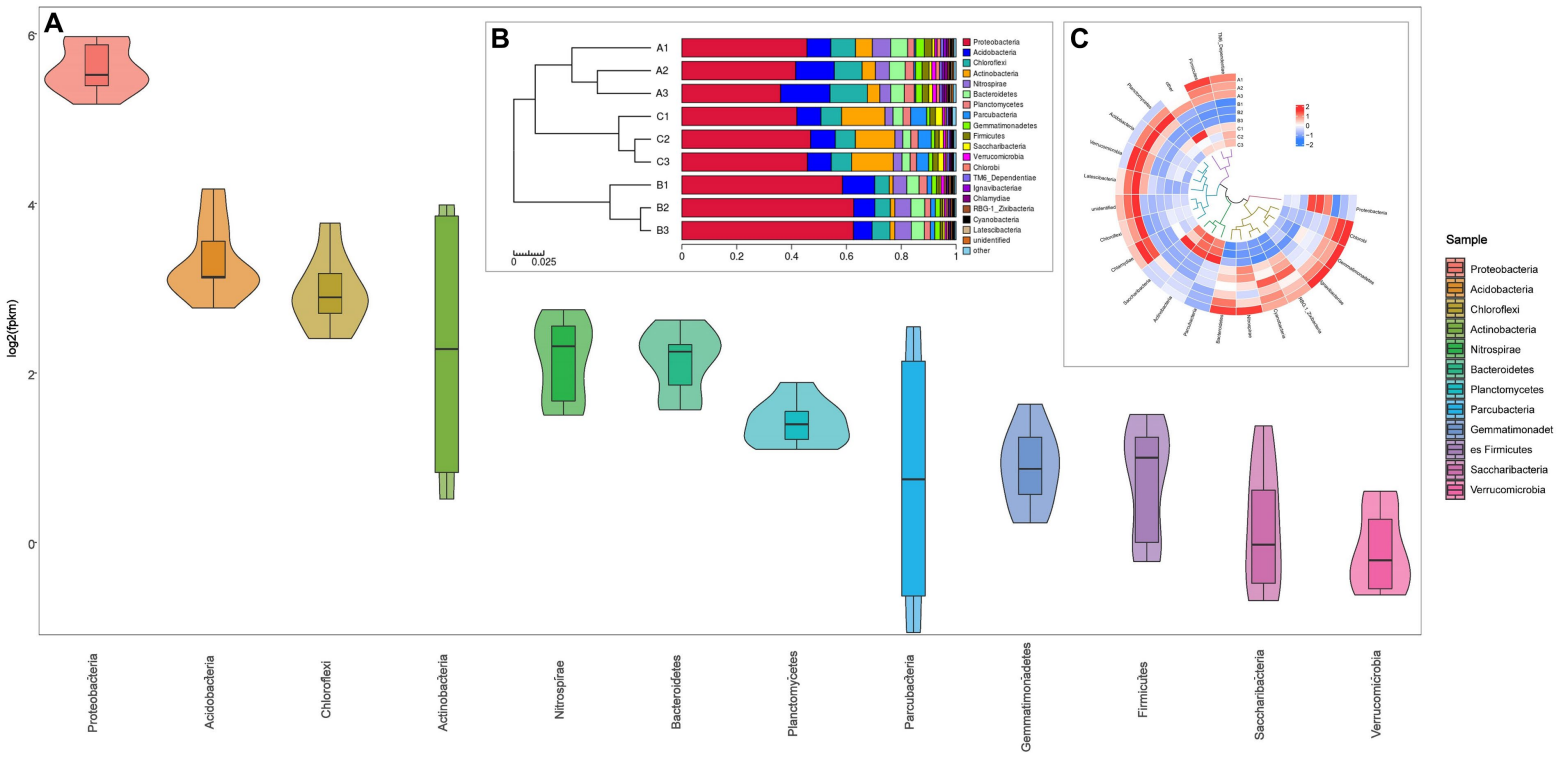
Relative abundance and composition of bacterial community at the phylum level. **(A)** the violin plots shows the distribution characteristic of different flora, of which the relative abundance of the total are more than 1%; **(B)** the clustered accumulation histograms shows the cluster characteristic between sampling groups; **(C)** the circle clustered heatmap shows the cluster characteristic between the flora in each sample.

The sequences derived from the specimens were subjected to analysis and subsequently organized into a total of 40 phyla, 111 classes, 143 orders, 263 families and 419 genera. The data presented in Figure 3 illustrates the distribution and prevalence of several specimens at the phylum level. The dominant phylum observed in the environment is *Proteobacteria*, accounting for 49.07% of the total composition. Other significant phyla include *Acidobacteria* (10.38%), *Chloroflexi* (8.05%), *Actinobacteria* (7.31%), *Nitrospirae* (4.62%), *Bacteroidetes* (4.53%). The relative abundance of *Proteobacteria* in samples A, O1, and O2 ranged from 36.01 to 45.6%, 58.57 to 62.66%, and 41.95 to 46.95%, respectively. The abundance of *Acidobacteria* ranged from 8.71 to 17.99%, 6.8 to 11.74%, and 8.69 to 9% in the respective samples. *Chloroflexi* exhibited an abundance of 9.01 to 13.64%, 5.3 to 6.51%, and 7.32 to 7.51%, respectively. The abundance of *Actinobacteria* ranged from 4.54 to 6.12%, 1.42 to 1.77%, and 14.41 to 15.81% in the respective samples. *Nitrospirae* exhibited abundances of 3.99 to 6.71%, 4.97 to 6.01%, and 2.96 to 3.62%, respectively. *Bacteroidetes* exhibited abundances of 4.93 to 6.16%, 4.52 to 5.04%, and 2.96 to 3.62% in the respective samples. When considering the unweighted Unifrac distances, an analysis of the microbial communities in cell A (representing the anoxic section), cells O1 and O2 (representing the aerobic section) revealed a statistically significant difference in the composition of flora between the aerobic sections. Based on compositional analysis, it was observed that *Proteobacteria* and *Nitrospirae* exhibited an initial increase followed by a subsequent decrease during the treatment process. Conversely, *Acidobacteria* and *Chloroflexi* displayed a decrease followed by an increase trend. The downward trend observed in *Actinobacteria* and *Bacteroidetes* is likely attributed to the microbial function of nitrification processes.

#### 3.2.2. Characterisation on the genus level

**Figure 4:**
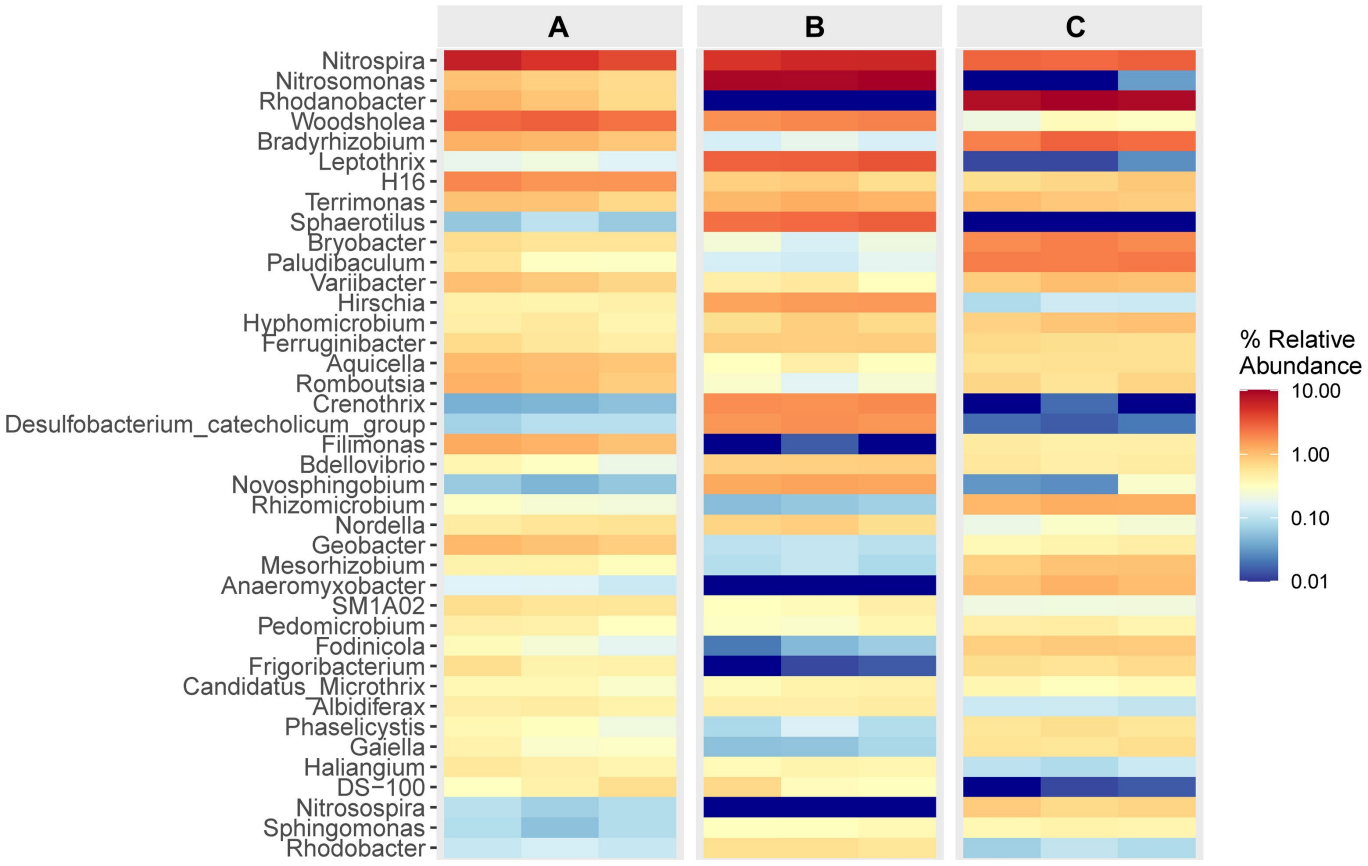
Relative abundance and composition of the top 40 bacterial community at the genus level. The colored heatmap shows the relative abundance and composition of flora from all specimens in different cells.

The data presented in Figure 4 illustrates the composition and relative abundance of the top 40 genera observed in each specimen. It is evident that in cell A, the prevailing genera were *Nitrospira* and *Woodsholea*, with respective abundances ranging from 3.91 to 6.67% and 2.42 to 3.04%. Similarly, in cell O1, the dominant genera were *Nitrosomonas*, *Nitrospira*, *Leptothrix*, with abundances ranging from 9.37 to 10.15%, 4.94 to 5.98%, and 2.98 to 3.44%, respectively. Likewise, in cell O2, the primary genera were *Nitrosomonas*, *Nitrospira* and *Leptothrix*, with abundances ranging from 8.68 to 9.99%, 2.67 to 3.03%, and 2.11 to 2.98%, respectively. The prevalence of the primary genera *Nitrospira* and *Woodsholea* in the effluent from the inlet of the WWT facility exhibited a consistent decline throughout the treatment process. In contrast, the abundance of *Nitrosomonas* and *Rhodanobacter* displayed significant variation, potentially indicating a correlation with the removal rate of contaminants that impact microbial growth. These findings further support the assumption that the WWT process significantly shapes the composition of the microbial community.

#### 3.2.3. Diversity of bacterial communities

**Figure 5:**
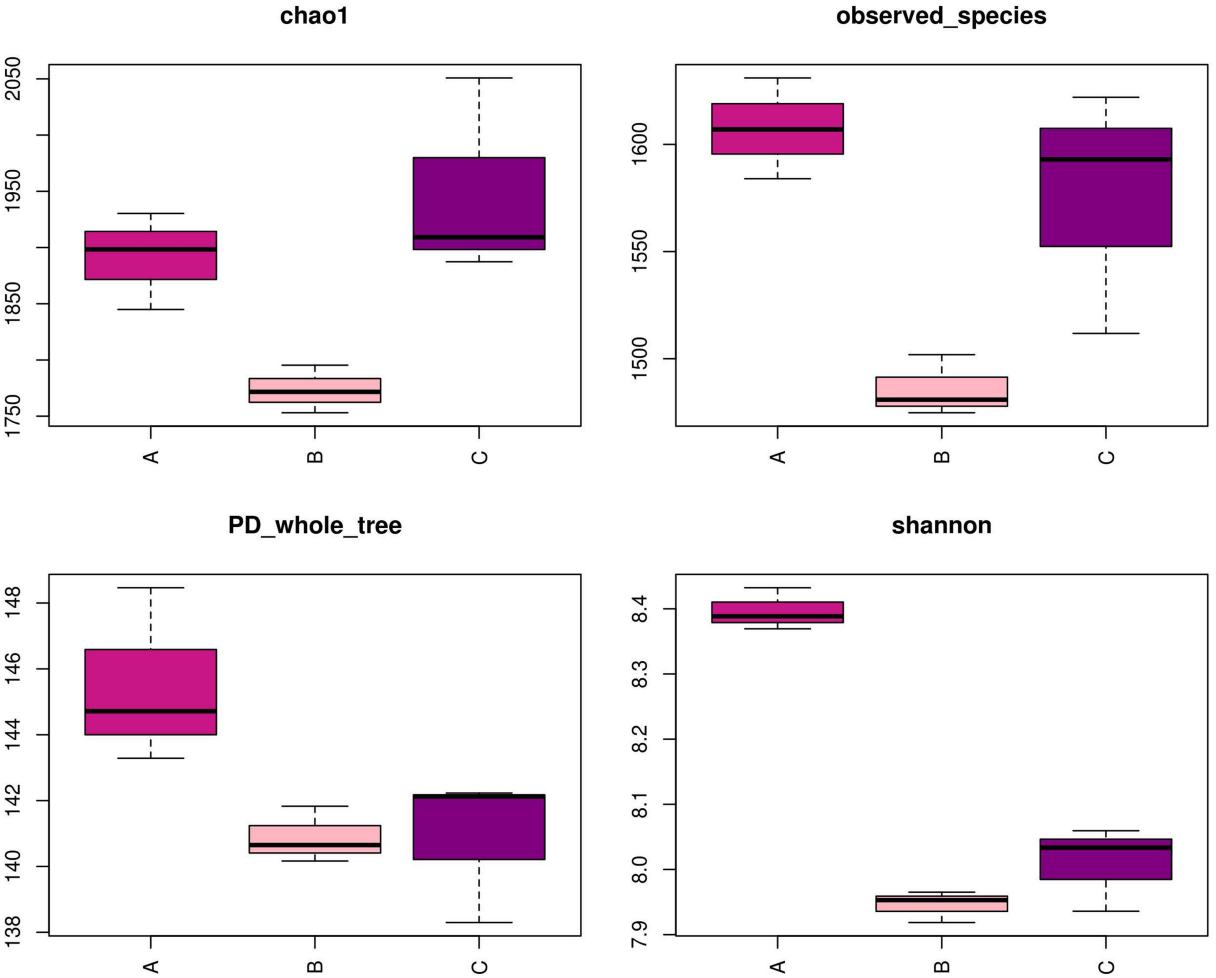
Diversity index in MMBF WWT. The box plots reflects the variation in the flora diversity in different cells by four diversity indicators.

Although the three compartments shared numerous predominant bacteria at the phylum level, the analysis at the genus level revealed a distinct community structure from OTUs, as depicted in Figure 5. Following the implementation of PCR and HTS, the number of OTUs observed in the nine samples exhibited a range of 1,475–1,631. Furthermore, the sequencing process achieved a coverage rate exceeding 97% for each sample, indicating the successful detection of the majority of microorganisms present in the samples. Notably, the Shannon index was employed to characterize community diversity, while observed species and the Chao1 index were utilized to evaluate community abundance (Fang et al., 2018). The calculation of those indicators reflected the assessment of variations in the microbial community structure. The findings of the study revealed that the Shannon index exhibited a statistically significant increase in cell A compared to the other cells, suggesting that cell A possessed the greatest microbial diversity. Additionally, the observed species and Chao1 index values obtained from cells A and O2 were significantly higher than those observed in cell O1, indicating an elevated microbial mortality in cell O1 and a rapid proliferation of the microbial community structure in cell O2.

### 3.3. Core divergent flora

**Figure 6:**
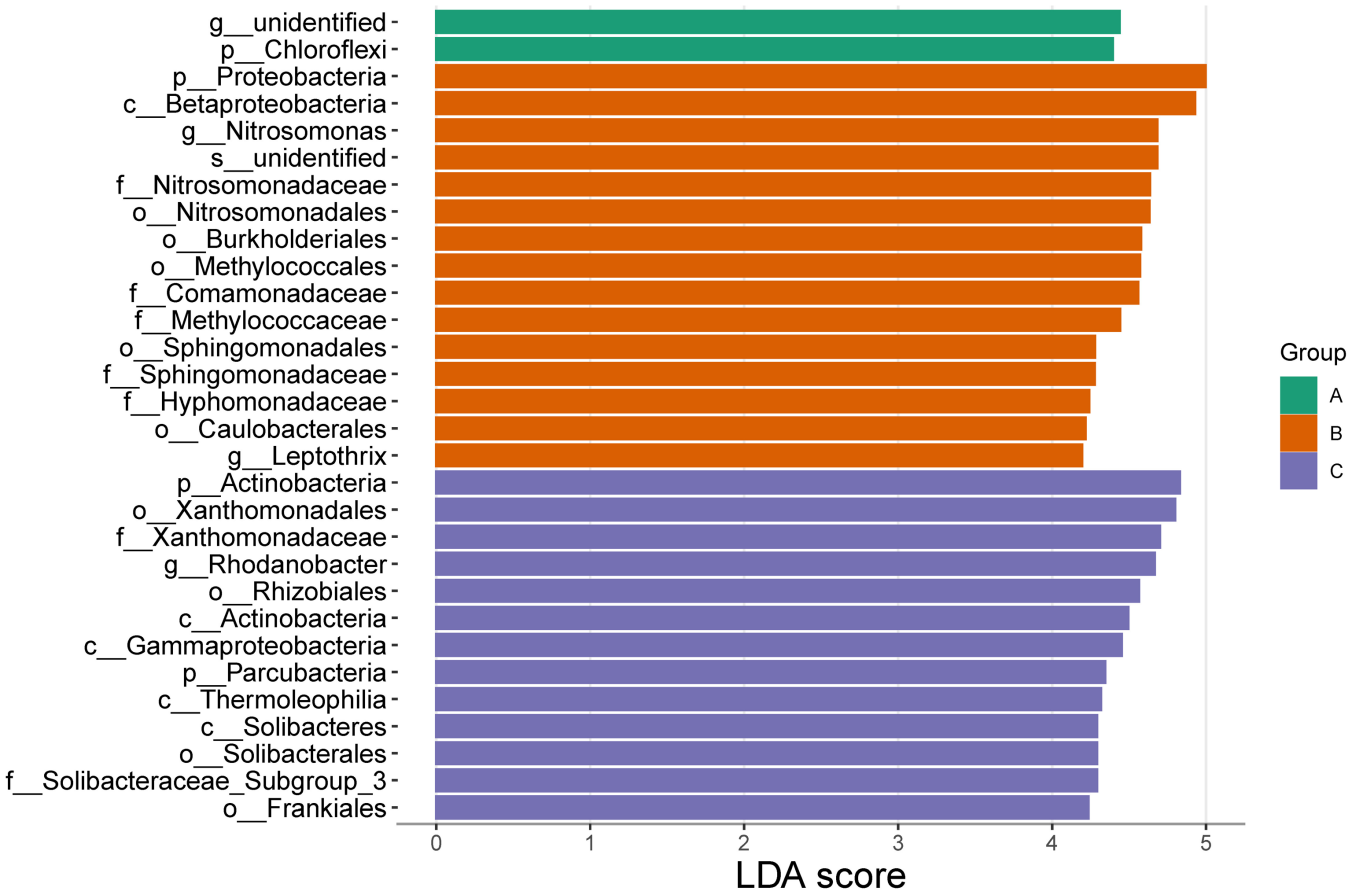
Significantly divergent species in MMBF WWT. The colored histogram reflects the dominant species screening by LDA score from the results of LEfSe.

**Figure 7:**
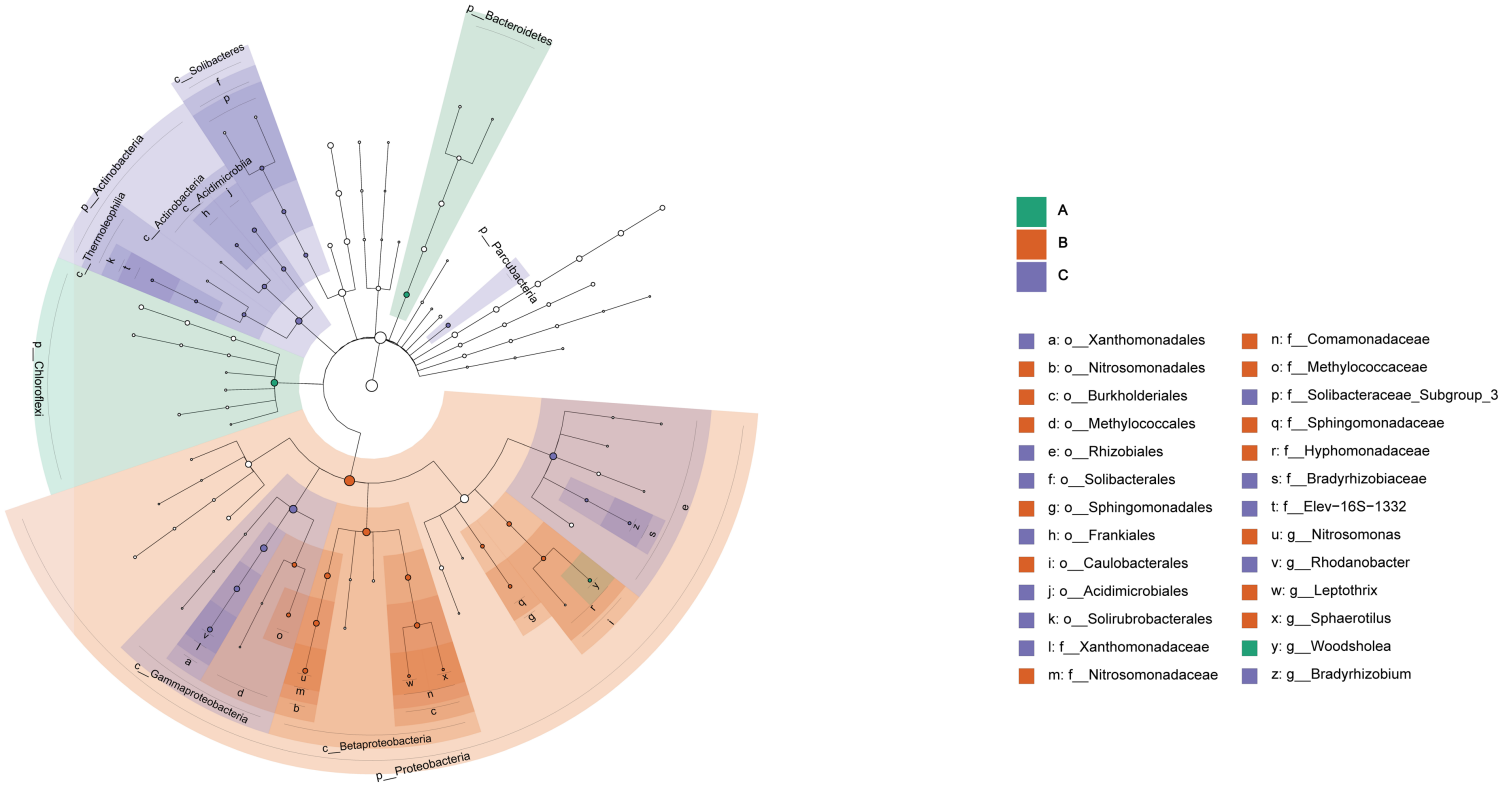
Significantly divergent levels of species impact in MMBF WWT. The figure shows circles radiating from the inside to the outside representing taxonomic levels from phylum to genus, with each node indicating a taxonomic unit at a different level, its diameter indicating the relative abundance size, and colored nodes indicating microbial taxa contributing significantly in the different groups.

Significant differences were found in the three cells through the analysis of community characteristics. To further analyze and identify the core differential communities, LEfSe analysis was performed on the samples at multiple taxonomic levels. Figure 6 displays a bar chart illustrating the distinct Biomarkers species that exhibit LDA scores more than 4 across sample groups. This figure provides insight into the influence of the considerably diverse species on cell A, O1, and O2. The visual representation in Figure 7 depicts a series of concentric circles that symbolize taxonomic levels ranging from phylum to genus. Each node within the circles represents a distinct taxonomic unit at a specific level, with the size of the node indicating its relative abundance. Additionally, nodes that are colored signify microbial taxa that make significant contributions within the various taxonomic groups. The findings indicate that there are notable variations in the MMBF treatment system across different groups, including *Chloroflexi*, *Woodsholea* (cell A), *Proteobacteria*, *Leptothrix*, *Caulobacterales*, *Hyphomonadaceae*, *Sphingomonadaceae Sphingomonadales*, *Sphingomonadales*, *Methylococcaceae*, *Comamonadaceae*, *Methylococcales*, *Burkholderiales*, *Nitrosomonadales*, *Nitrosomonadaceae*, *Nitrosomonas Betaproteobacteria* (cell O1), *Actinobacteria*, *Frankiales*, *Solibacteraceae_Subgroup_3*, *Solibacterales*, *Solibacteres*, *Thermoleophilia*, *Parcubacteria*, *Gammaproteobacteria*, *Actinobacteria*, *Rhizobiales*, *Rhodanobacter*, *Xanthomonadaceae*, *Xanthomonadales* (cell O2).

### 3.4. Function of the bacterium community

**Figure 8:**
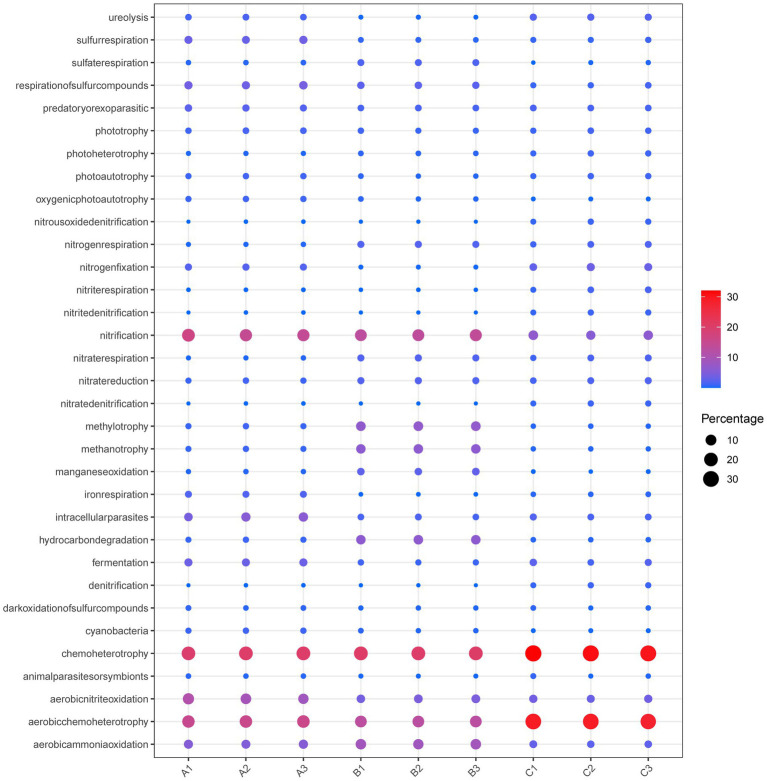
Differences in predicted gene function of bacterial populations in MMBF WWT. The bubble plot shows the differences in the distribution of functional nitrogen removal genes associated with the nitrogen cycle between different specimens.

The findings presented in Figure 8 demonstrate variations in predicted functional genes across different samples. The predominant bacterial community gene functions observed in all samples were chemoheterotrophy (23.9%), aerobic chemoheterotrophy (17.97%), nitrification (11.13%), aerobic ammonia oxidation (5.95%), aerobic nitrite oxidation (5.18%), and so forth, comprising 65 gene functions. Among the microbial communities analyzed, various metabolic processes were observed to contribute to the overall gene abundance in different cells. Specifically, chemoheterotrophy, aerobic chemoheterotrophy, nitrification, aerobic ammonia oxidation, and aerobic nitrite oxidation accounted for different proportions of the total gene abundance in cell A, cell O1, and cell O2. In cell A, these processes represented 19.58, 14.85, 14.78, 5.27, and 9.51% of the total gene abundance, respectively. In cell O1, the proportions were 19.93, 12.38, 13.03, 8.58, and 4.45%, respectively. In cell O2, the proportions were 29.95, 27.76, 6.01, 2.55, and 3.46%, respectively. Notably, the effluent treatment process had a significant impact on the prevalence of chemoheterotrophy, as significantly increased, while nitrification and aerobic nitrite oxidation gradually decreased. These findings suggest that the relevant gene functions associated with these metabolic processes play a crucial role in the WWT process.

The distribution patterns of functional nitrogen removal genes related to the nitrogen cycle indicate that cell A predominantly contains nitrification and aerobic nitrite oxidation processes. On the other hand, aerobic ammonia oxidation, nitrate reduction, and nitrogen fixation processes are primarily associated with cell O1. Additionally, cell O2 is primarily responsible for nitrogen fixation, nitrite respiration, and denitrification processes (including nitrate denitrification, nitrite respiration, and the reduction of nitrates and nitrites). Therefore, it is assumed that there exist six distinct routes involved in nitrogen metabolism, namely nitrification, denitrification, assimilative nitrate reduction, dissimilative nitrate reduction, anammox, and nitrogen fixation.

## 4. Discussion

The findings from the analysis of the microbial community identified a total of 40 phyla, 111 classes, 143 orders, 263 families, and 419 genera of bacteria. The predominant phylum observed in each cell of the process exhibited similarities to previous studies conducted on urban WWT plants, specifically *Proteobacteria, Acidobacteria*, *Chloroflexi*, *Actinobacteria*, *Nitrospirae*, *Bacteroidetes* (Yin et al., 2019; Yasir, 2020; Zhang et al., 2020; Rodriguez et al., 2021). In addition, the number of species in each levels is similar with those in sludge of a WWT plants during winter in Xinjiang, China (Xu et al., 2018), as well as the dominant phyla shared with activated sludge are *Proteobacteria*, *Chloroflexi*, *Acidobacteria*. The composition of the microbial community in the WWT system exhibits a strong correlation with the influent substrate concentration (Pang et al., 2015; Zhang et al., 2019b). Those finding implies that the microbial community composition of decentralized MMBF WWF is comparable to those in conventional WWT plants during cold weather. Notably, the WWT system harbors a diverse and intricate microbial community that adheres to the carrier surface, playing a crucial role in the WWT process.

Through long-term monitoring of the water quality treatment effect, it revealed the process has high removal efficiency of COD, TN, NH_3_-N and TP in the effluent on average, and has a high capacity of surge resistance, with good treatment effect and stable effluent quality. In conjunction with the physical and chemical indicators at the inlet and outlet, it can be inferred that the observed proliferation of bacterial species is mostly attributed to their consumption of COD, NH_3_-N, TN and TP. This inference is supported by the observed declining trends of COD, NH_3_-N, TN, and TP throughout the process flow.

As aimed to examine the spatial distribution of microbial communities in the treatment system and analyze the nitrogen removal pathways along the system, our findings revealed that the anoxic cell made the most substantial contribution (68.36%) to TN removal. Notably, anoxic cell displayed the highest microbial population diversity, with the most abundant genus identified in cell A, O1, and O2 were *Nitrospira*, *Nitrosomonas*, and *Rhodanobacter*, respectively. Those finding suggests that different microbial community diversity between anoxic cell and aerobic cells contributes to the removal of TN, NH_3_-N in different process through nitrogen removal pathways.

Based on relevant research, *Proteobacteria* are the main species involved in the degradation of contaminants including bio-denitrogenation and phosphorus removal (Huang et al., 2018). This phylum typically exhibits dominance in urban domestic wastewater settings, as well as in industrial wastewater, including tannery wastewater, and aquaculture water environment (Rose et al., 2020; Jankowski et al., 2022). The *Acidobacteria* phylum is prevalent in anaerobic ammonia-oxidizing systems and possesses the functional gene nosZ to participate in denitrification (Yang et al., 2021). The abundance of *Acidobacteria* is significantly affected by pH and appropriate for existence in conditions with low pH (Zhang et al., 2019b; Xie et al., 2021). *Chloroflexi* has been observed to engage in the process of decomposing organisms within deceased cells (Zhao et al., 2018). Additionally, it has been found to exhibit a preference for metabolizing deceased aerobic ammonia-oxidizing bacteria (AAOB) within anaerobic ammonia-oxidizing systems (Nierychlo et al., 2018), and promote accumulation of AAOB to avoid accumulation of organic waste (Gu et al., 2022). *Actinobacteria* (Tilahun, 2020) primarily contribute to the degradation of organism, significantly correlated with COD and BOD5, and the abundance is positively correlated with the occurrence of sludge inflation (Jankowski et al., 2022). *Nitrospirae* plays a crucial role in biological denitrification (Zhang et al., 2019b), as the primary nitrite oxidizing bacterium, and is commonly found in urban WWT systems throughout various seasons. Notably, nitrification activity is typically hindered under cold conditions (Zhou et al., 2018). However, contrary to this trend, the relative abundance of *Nitrospirae* is higher at lower temperatures (Luo et al., 2020). *Bacteroidetes* can degrade organic contaminants in wastewater, and can carry functional genes norB and nosZ to participate in denitrification, which are also commonly observed in anammox systems associated with efficient organic matter degradation, as well as fostering sludge (Yang et al., 2020b). *Nitrospira* is the major nitrite oxidizing bacterium found in WWT plants, converting nitrite to nitrate with a better denitrification capacity as compared to *Nitrobacter* (Rose et al., 2020). *Nitrosomonas* is commonly known as an ammonia-oxidizing bacterium, capable of oxidizing ammonia to nitroso-nitrogen in influent water, co-dominant with anaerobic ammonia-oxidizing bacteria to TN removal, and has denitrification-related genes (Wang et al., 2019).

The core differential organisms were subjected to further analysis and identification using the LEfSe method. The process of denitrogenation holds great importance within the WWT system, and the functional genes responsible for nitrogen metabolism play a critical role in this context (Yin et al., 2022). FAPROTAX method provides the investigation of gene abundance in the nitrogen cycle elucidates the presence of six distinct pathways involved in nitrogen metabolism. These routes primarily encompass nitrification, denitrification, anabolic nitrate reduction, anisotropic nitrate reduction, anammox, and nitrogen fixation. The prevalence of nitrification genes within the system surpasses that of other genes associated with the nitrogen cycle, such as denitrification genes. In addition, the assimilatory nitrate reduction and anabolic nitrate reduction pathways encompass a range of ammonia genes. These genes facilitate the conversion of nitrate to nitrite and ammonia, with the latter being assimilated into amino acids to support the synthesis of essential cellular components. Furthermore, anabolic nitrate reduction, which occurs in microorganisms during oxygen-deprived conditions, involves the process of nitrate respiration. Despite the relatively low prevalence of anaerobic genes within the system, certain dominant microbial taxa such as *Acidobacteria* and *Chloroflexi* have been found to participate in anammox. This finding partially elucidates the potential occurrence of the anammox process, which facilitates the rapid degradation of total nitrogen in oxygen-depleted compartments. In the denitrification process, denitrification plays a crucial role in reducing nitrate nitrogen to nitrite nitrogen (Wang et al., 2019). Notably, the dominant microbial taxa *Acidobacteria*, *Bacteroidetes*, *Nitrospira*, and *Nitrosomonas* possess the necessary genetic elements for denitrification, indicating the system’s favorable denitrification performance. Moreover, it is important to acknowledge that maintaining the optimum temperature is crucial for the effectiveness of biochemical treatment (Gu et al., 2022). This can provide challenges in meeting emission regulations during the winter season. *Nitrospirae*, *Nitrospira* and other prevalent microbial communities could acclimate to low temperature environment and actively engage in nitrification processes. These organisms serve as the primary denitrification agents in cold conditions. The findings of this study provide confirmation that the MMBF exhibits a notable level of efficiency as a decentralized WWT facility for the purpose of nitrogen removal. This indicates that the MMBF has the potential for broad application in various settings, such as highway service areas and water service areas.

## 5. Conclusion

This study investigated the microbial community characteristics of a typical decentralized WWT system in expressway service area. The process appears with high removal efficiency of COD, TN, NH_3_-N and TP in the effluent on average. The results of the microbial community identification revealed 40 phyla, 111 classes, 143 orders, 263 families and 419 genera of microorganisms. The predominant phylum observed are *Proteobacteria, Acidobacteria*, *Chloroflexi*, *Actinobacteria*, *Nitrospirae*, *Bacteroidetes*. There are six nitrogen metabolism pathways, primarily nitrification, among which *Nitrospirae* and *Nitrospira* are the core differentiated flora participate in nitrification, as the dominant nitrogen removal flora in cold regions. The microbial community composition of decentralized MMBF WWF is similar to those in conventional WWT plants during cold weather. Those results confirm that the MMBF system has achieved high performance in nitrogen removal. This study provides theoretical and data support for the construction of efficient decentralized wastewater biological treatment systems.

## Data availability statement

The raw data supporting the conclusions of this article will be made available by the authors, without undue reservation.

## Author contributions

XL, YK, and SH designed the study and experiments. SH performed the bioinformatics analyses and wrote the manuscript. SH, XL, and YC analyzed the data. XH and PM performed the sample collection and experiment. SH, XL, and YK edited the manuscript. All authors contributed to the article and approved the submitted version.

## Footnotes

^1^ https://www.cloudtutu.com
